# Dietary Fat Chain Length, Saturation, and PUFA Source Acutely Affect Diet-Induced Thermogenesis but Not Satiety in Adults in a Randomized, Crossover Trial

**DOI:** 10.3390/nu13082615

**Published:** 2021-07-29

**Authors:** Bret M. Rust, Susan K. Raatz, Shanon L. Casperson, Sara E. Duke, Matthew J. Picklo

**Affiliations:** 1 USDA-ARS Grand Forks Human Nutrition Research Center, Grand Forks, ND 58203, USA; brust@ucdavis.edu (B.M.R.); raatz001@umn.edu (S.K.R.); shanon.casperson@usda.gov (S.L.C.); 2 Department of Nursing, University of North Dakota, Grand Forks, ND 58202, USA; 3 Department of Food Science and Nutrition, University of Minnesota, Minneapolis, MN 55108, USA; 4 USDA-ARS-PA-NRRC, Fort Collins, CO 80526, USA; sara.duke@usda.gov; 5 Department of Chemistry, University of North Dakota, Grand Forks, ND 58202, USA

**Keywords:** diet-induced thermogenesis, satiety, polyunsaturated fatty acids, thermic effect of food, fat oxidation, respiratory exchange ratio

## Abstract

Structural differences in dietary fatty acids modify their rate of oxidation and effect on satiety, endpoints that may influence the development of obesity. This study tests the hypothesis that meals containing fat sources with elevated unsaturated fats will result in greater postprandial energy expenditure, fat oxidation, and satiety than meals containing fats with greater saturation. In a randomized, 5-way crossover design, healthy men and women (*n* = 23; age: 25.7 ± 6.6 years; BMI: 27.7 ± 3.8 kg/m^2^) consumed liquid meals containing 30 g of fat from heavy cream (HC), olive oil (OO), sunflower oil (SFO), flaxseed oil (FSO), and fish oil (FO). Energy expenditure and diet-induced thermogenesis (DIT) were determined by metabolic rate over a 240 min postprandial period. Serum concentrations of ghrelin, glucose, insulin, and triacylglycerol (TAG) were assessed. DIT induced by SFO was 5% lower than HC and FO (*p* = 0.04). Energy expenditure and substrate oxidation did not differ between fat sources. Postprandial TAG concentrations were significantly affected by fat source (*p* = 0.0001). Varying fat sources by the degree of saturation and PUFA type modified DIT but not satiety responses in normal to obese adult men and women.

## 1. Introduction

Dietary fat comprises more than 30% of energy intake for Americans, and epidemiological studies show positive correlations between energy from total fat intake and obesity [1,2,3,4,5]. Epidemiological evidence reveals that differences in macronutrient intake, including fat sources, may account for differences in risk for cardiovascular disease and obesity [6]. Clinical and mechanistic studies have expanded our understanding of how dietary fatty acids (e.g., saturated fatty acids (SFA), monounsaturated fatty acids (MUFA), and polyunsaturated fatty acids (PUFA)) elicit divergent physiologic effects that impact energy expenditure and energy intake (via satiety), major factors influencing body weight [7].

Differences in the chain length and the degree of saturation of fatty acids comprising dietary fat may affect fat accumulation by altering thermogenesis. The thermic effect of food or diet-induced thermogenesis (DIT), defined as the postprandial increase in energy expenditure, is estimated to account for up to 10% of daily energy expenditure [8]. It is documented that medium chain SFA present in medium chain triglycerides are preferentially oxidized and elevate energy expenditure vs. longer chain SFA [9,10]. However, findings from studies of postprandial energy expenditure comparing dietary oils containing higher contents of SFA, PUFA, and/or MUFA, typically oleic acid (OA; 18:1*n*-9), have varied [11,12,13,14,15,16].

Although tracer studies in humans indicate that the PUFA alpha-linolenic acid (ALA; 18:3*n*-3) undergoes a higher rate of oxidation vs. linoleic acid (LA; 18:2*n*-6), most studies, except for those of Casas-Augustench and colleagues and Jones and colleagues, have used LA-containing oils or a combination of ALA-containing and LA-containing oils as a PUFA source when studying postprandial energy metabolism [11,12,13,16,17,18]. The commercial availability of oils naturally possessing elevated concentrations of ALA, LA, and long chain *n*-3 PUFA (LCn-3), such as docosahexaenoic acid (22:6*n*-3) and eicosapentaenoic acid (20:5*n*-3), point to a need to determine the influence of PUFA type (ALA vs. LA vs. LCn-3) upon postprandial energy expenditure and substrate utilization.

In addition to potential modulation of DIT, the fatty acid composition of dietary fats may influence food intake or energy intake and satiety. Studies designed to compare the impact of saturation indicate that fats containing unsaturated 18-carbon fatty acids (LA and OA) increase subjective measures of satiety (SMS) and/or reduce food intake or energy intake in a subsequent meal (ESM) compared to fat sources containing stearic acid (18:0) [19,20]. Studies using dairy fat as a source of SFA have not reported differences between SFA, MUFA, and PUFA upon satiety and subsequent food intake and energy intake; whereas SFA sources, containing 16:0 and 12:0 derived from a combination of dairy, coconut oil, and palm kernel oil, provide greater satiety than MUFA or PUFA [16,21,22,23]. Similar to the existing literature regarding energy expenditure, there is a paucity of data regarding the impact of PUFA type (ALA vs. LA vs. LCn-3) upon SMS and ESM.

The aim of this work was to address the role of fat sources naturally high in specific dietary fatty acids in regulating satiety and energy metabolism. This research used a systematic approach to determine the impact of saturation and chain length of fat sources on energy metabolism and satiety in healthy individuals by comparing heavy cream (HC) as a source of SFA, olive oil (OO) as a source of OA, sunflower oil (SFO), and flaxseed oil (FSO) as sources of LA and ALA, respectively, and a fish oil (FO) product as a source of LCn-3. We hypothesized that meals containing unsaturated fats have greater postprandial energy expenditure, fat oxidation, and satiety than meals containing fats with greater saturation. As mechanistic endpoints, we determined the influence of dietary fat on postprandial plasma responses of the satiety hormone ghrelin, glucose, insulin, and triacylglycerol (TAG).

## 2. Methods

### 2.1. Study Design and Intervention

This study was a single-center, randomized, five-way crossover design trial developed to evaluate the acute postprandial effects of liquid test meals prepared with fats of different fatty acid compositions with regard to chain length and degree of saturation. The test fat sources were either HC, OO, SFO, FSO, or FO. HC was selected as a source of SFA because dairy is a major source of dietary SFA in America, and HC facilitates the use of liquid meals [24]. Sequences of the test fat source treatments were developed using a William’s 5-way crossover design model [25]. Participants were block randomized to assure balanced assignment. The treatment assignments were generated by a USDA-ARS statistician. The randomization protocol is provided in Supplementary Table S1. A washout period of at least seven days and a three-day standard lead-in diet were provided prior to test visits in order to minimize carryover and potential effects of the participant’s habitual diet. The investigators and the participants were blinded to the test fat provided.

Test visits were conducted in a whole room calorimeter overnight followed by the intervention the following morning. The intervention consisted of ingesting the liquid test meal with measures of energy expenditure and satiety and blood sampling was performed prior to and for 240 min after ingesting the test meal. An ad libitum meal was subsequently provided to determine ESM. Test visits were separated by at least 7 days. All study visits were conducted at the USDA-ARS Grand Forks Human Nutrition Research Center (GFHNRC). Written, informed consent was provided by all participants prior to beginning the study procedures. Approval for the study was obtained from the University of North Dakota Institutional Review Board (protocol code 201506-376, approved 11 June 2015). The study was registered at www.ClinicalTrials.gov (NCT02496936).

### 2.2. Participants

Participants were recruited with newspaper advertisements, fliers, and e-mail announcements distributed within the University of North Dakota and the Grand Forks, ND area. Recruitment began in January 2016 and was concluded in October 2019 when the recruitment goals were met. Data were collected and analyzed from 23 participants (11 women, 12 men) who completed all five test treatments. Initial study criteria included women aged 18 to 50 years with a body mass index (BMI) between 30.0–34.9 kg/m^2^ and free of major medical conditions. Due to low recruitment, study criteria were revised to include men and women aged 18 to 50 years, body mass index between 18 and 34.9 kg/m^2^, and free of major medical conditions. Exclusion criteria included: smoking and nicotine use, steroid use other than oral contraceptives for women, pregnancy or lactation, diagnosed eating disorder, diabetes, diagnosed cardiovascular, pulmonary, skeletal, and metabolic diseases, use of medications known to affect appetite, blood lipids, body composition, body weight, or food intake (appetite control drugs, steroids, or antidepressants), women not using oral hormonal contraception, and high LCn-3 intake measured by the “Omega-3 checklist” [26]. Participants were excluded if they had lost > 10% of their weight in the two months preceding screening and/or were actively attempting weight loss.

Applicants were screened with an online application or telephone interview. Applicants who met the initial screening criteria from self-reported data were invited to an informational meeting in which the study personnel described the study in detail and answered any questions about the study procedures. Height, body mass, blood pressure, and fasting blood glucose by fingerstick (Accu-Chek Compact Plus, F. Roche Diabetes Care, Inc., Indianapolis, IN, USA) were measured to assess eligibility. The participants’ usual dietary intake was determined by completion of the web-based Diet History Questionnaire II (DHQ II) (http://appliedresearch.cancer.gov/dhq2/).

**Figure 1:**
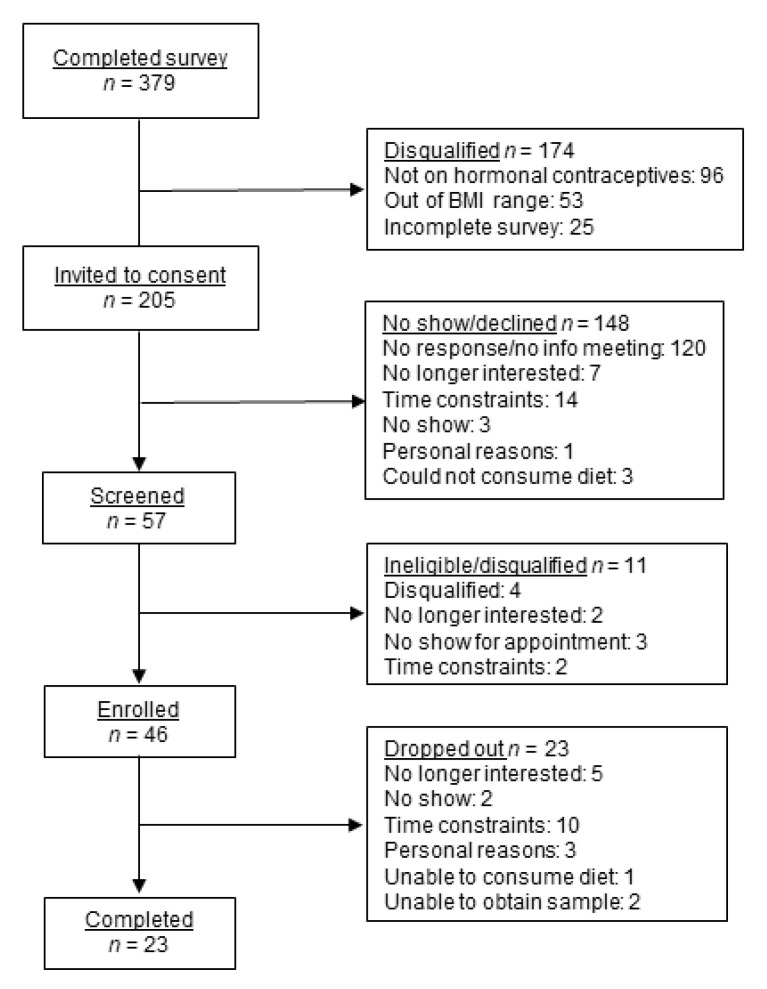
CONSORT diagram indicating the number of participants completing the initial study survey, invited to an information meeting for consent, screened, enrolled, randomized into a treatment order, and completing each of the five treatment arms.

Body mass was measured (to 0.1 kg) using a calibrated digital scale (Model 50735, Fairbanks Scales, Kansas City, MO, USA) with subjects wearing light clothing without shoes. Stature (to 1 mm) was measured with a free-standing stadiometer (Model S-214, Seca, Hamburg, Germany). A medical history questionnaire was used to determine medical eligibility. A CONSORT diagram (Figure 1) provides recruitment, consents, dropouts, and completion information.

### 2.3. Study Implementation

#### 2.3.1. Whole Room Calorimetry

Specifications of the calorimeter at the GFNRC are published [27]. Briefly, the calorimeter operates in a “push-pull” configuration with a total volume of 25,000 L. Each mass flow controller (MFC) is rated for an accuracy of 1% full scale according to the manufacturer’s specifications but are regularly calibrated to obtain a 0.5% accuracy reading for each MFC. System performance is regularly checked using blended infusions that mimic human physiological states. System calibration was confirmed prior to each study visit with a coefficient of variation (CV) < 1%. The mean within subjects’ CV for the calorimeter was 1.85% for VO_2_, 2.04% for VCO_2_ which resulted in a mean CV for EE of 1.90% and a mean CV for RQ of 1.32%. The mean CV for the resting metabolic rate (RMR) for the visits within this study was 6.4%.

#### 2.3.2. Pretest Procedures

Following the provision of consent and screening, eligible participants were scheduled for overnight stays in the whole room calorimeter. Prior to admission to the GFHRNC, a three-day, isocaloric, lead-in diet was provided to control for the potential effects of the participants’ usual diet (see below). Total energy needs were estimated in order to calculate the energy required for the lead-in diet. In order to determine energy needs, resting energy expenditure (REE) was measured with indirect spirometry on a metabolic cart (TrueOne Metabolic Monitor, Parvomedics, Sandy, UT, USA). REE was multiplied by an activity factor ascertained from a validated questionnaire (Stanford Brief Activity Survey) [28]. Participants were instructed to refrain from exercise or alcohol intake for 72 h prior to admission to each study visit.

All participants were admitted to the GFHRNC metabolic unit no later than 5 p.m. the evening prior to each test day. Following admission and body weight measurement, participants consumed the final dinner meal of the lead-in diet before 7 p.m. and entered the whole room calorimeter before 9 p.m. Lights in the calorimeter were turned off at 11 p.m. Respiratory gas exchange data were collected the entire time participants were in the whole room calorimeter.

#### 2.3.3. Test Day Protocol

Participants were awakened at 6 a.m. on the test day and were allowed 15 min for morning toilette and urinary void before undergoing a 30-min RMR measurement. For the RMR measurement, participants lay awake and without activity, including reading and the use of electronic devices. Following the RMR, participants were removed from the calorimeter for insertion of an indwelling catheter into the antecubital vein after which they were returned to the calorimeter.

Following re-entry into the calorimeter, participants sat comfortably in a reclining chair adjacent to a blood draw port in the door of the calorimeter. After the gas exchange was stable for at least five minutes a fasting blood draw was taken through the blood draw port at approximately 7:30 a.m. The participants completed a computer-mediated, four-question satiety questionnaire using the Sussex Ingestion Pattern Monitor (Sussex Innovation Center, Brighton, UK) software. Immediately following the satiety questionnaire, a liquid meal containing one of the test fats was provided through a passthrough adjacent to the calorimeter door and was consumed by the participant within 15 min of the fasting blood draw. Participants remained in the reclining chair for the duration of the 240-min postprandial period except to use the bathroom. The calorimeter detected and recorded movement with an infra-red motion detector. Blood draws and follow-up satiety questionnaires were taken at 30, 60, 120, 180, and 240 min following the fasting blood draw. Following the final blood draw, the catheter was removed through the blood draw port and participants were free to move about the calorimeter until 300 min from the fasting blood draw. After 300 min, participants exited the calorimeter and consumed a standard ad libitum meal (see below) to determine ESM. The participants remained within sight of the study personnel for the duration of the visit to the metabolic unit of the GFHNRC.

### 2.4. Meal Compositions

All meals were prepared in the GFHNRC metabolic kitchen. The three-day, lead-in diet provided 15% energy (en) protein, 50% en carbohydrate, and 35% en fat with SFA:MUFA:PUFA ratio of 1:1:1 (Supplementary Table S2).

The calculated nutrient composition for each liquid test meal is illustrated in Table 1. Each liquid test meal contained 30 g of one of the experimental fats, and this amount of fat was chosen as the dose to equal or exceed the amount used in other acute interventions [14,15,16]. Test meals provided SFA from heavy cream (Land O Lakes, Whitewave Foods, Broomfield, CO, USA), the MUFA, oleic acid (18:1*n*-9), from olive oil (Natural Oils International, Inc., Simi Valley, CA, USA), linoleic acid (LA; 18:2*n*-6) from sunflower oil (A&M Gourmet Foods, Inc., Toronto, ON Canada), alpha-linolenic acid (ALA; 18:3*n*-3) from flaxseed oil (Spectrum Organic Products, LLC—Subsidiary of the Hain Celestial Group, Inc., Melville, NY, USA), and LCn-3 from fish oil (The Coromega Company, Vista, CA, USA). Other ingredients comprising the shakes were frozen whole strawberries (Our Family, Minneapolis, MN, USA), orange sherbet (Our Family, Minneapolis, MN, USA), and skim milk (Cass Clay, Fargo, ND, USA). The final energy distribution of the liquid test meals was approximately 4% en from protein, 35–39% en from carbohydrate, and 57–62% en from fat. Variations in % en distribution were due to the heavy cream and fish oil matrices used as sources of SFA and LCn-3, respectively.

The calculated energy content ranged from 456 to 517 kcal; the percentage of daily needs (±SD) calculated from the resting energy expenditure and activity level provided by the liquid test meals averaged 16.2 ± 3.1% en (OO meal), 16.3 ± 3.1% en (FSO meal), 15.9 ± 3.0% en (SFO meal), 16.9 ± 3.2% en (HC meal), and 18.1 ± 3.4% en (FO meal). The fatty acid compositions of the liquid test meals were determined by fatty acid methyl ester (FAME) analysis and are provided in Supplementary Table S3.

The standard post treatment ad libitum meal consisted of a chicken noodle casserole containing 24.5% en protein, 37.5% en carbohydrate, and 38% en fat (Supplementary Table S4). This meal was weighed before and after consumption to calculate the ESM.

### 2.5. Fatty Acid Determination of Liquid Meals

Test meals were freeze-dried to determine water content. The dried component was homogenized to an even consistency using the Stomacher paddle mixer (Seward, West Sussex, UK). A 0.5 g portion of shake solids was extracted using the method of Folch and colleagues [30]. The solvent was removed and the oil residue was weighed for gravimetric determination of fat content. The oil was analyzed by fatty acid methyl ester analysis as previously published [31]. Each sample was analyzed in triplicate for oil content and fatty acid composition.

### 2.6. Urine Collection and Analysis

Urine samples were collected over the course of the postprandial period following a morning void. At the end of the postprandial period, the collections were pooled and the specific gravity and volumes recorded. Urine was aliquoted, frozen, and stored for nitrogen content analysis. Total nitrogen was determined using the Dumas combustion method (Rapid N Exceed; Elementar Americas Inc., Mt. Laurel, NJ, USA).

### 2.7. Blood Collection

Blood was collected at baseline and 30, 60, 120, 180, and 240 min from fasting through an indwelling catheter. Serum tubes were allowed to clot at room temp for at least 30 min, and all tubes were centrifuged at 3000× rpm at 4 °C for 10 min. Serum insulin concentration was determined using the IMMULITE 1000 System (Siemens Healthcare, Llanberis, Gwynedd, UK) with the insulin kit (cat# LKIN1). Serum ghrelin concentration was measured with an ELISA-based assay (Cat# EZGRA-88K; Waters-Millipore, Milford, MA, USA). Serum concentrations of glucose and triacylglycerol were measured by the COBAS INTEGRA 400 PLUS (Roche Diagnostics, Indianapolis, IN, USA) with the Glu HK Gen.3 kit (cat# 04404483190) for glucose and TRIGL kit (cat# 20767107322) for triacylglycerol.

### 2.8. Data and Statistical Analyses

The 23 subjects needed were estimated based on a repeated-measures analysis of variance (ANOVA) design; estimates of the within-individual subject variation were obtained from previous studies [16,20,32]. Determination of sample size was based on 90% power to detect a difference between any two means for our primary outcome of energy expenditure as measured by the metabolic rate. Twenty participants were needed to detect a 29% change in DIT with a within-subject SD of 2% [16]. For β-oxidation, assuming a within-subject SD 0.14 kcal/min, 23 subjects were needed to detect a difference of 0.17 kcal/min [32]. Assuming a within-subject SD of 10 mm x min area under the curve (AUC) in hunger, it was determined that 23 participants would be needed to detect a difference of 12 mm x min between any of the dietary fat types [20].

In two visits for two different participants, fasting TAGs were found to be highly elevated and subsequent postprandial TAG declined suggesting non-compliance with the lead-in diet and/or alcohol abstinence. The SFA meal was consumed at these visits. Data from those visits were excluded from our analysis. A leak in the arm port during one participant’s stay made metabolic data from that visit unusable. This also occurred with the SFA meal. One participant had elevated insulin concentrations at the final two visits in which this participant’s fasting insulin was greater than 17 standard deviations more than the overall mean fasting insulin for both of those test days. Insulin data for that participant from those visits were removed from further analysis.

Raw gas exchange data were collected as previously described [27]. Briefly, a null offset was employed to correct for any differences in the gas analyzers. Minute-by-minute VO_2_ and VCO_2_ data were uploaded into the PiLR proprietary software (MEI Research Inc., Edina, MN, USA) to calculate the metabolic rate, DIT, non-protein respiratory exchange ratio, and carbohydrate and fat oxidation rates at specific intervals during the test day protocol: 45–60, 90–105, 150–165, and 210–225 min following the fasting blood draw. When activity counts registered by the activity monitors corresponded to increases in average oxygen consumption (VO_2_) and carbon dioxide production (VCO_2_), those data were excluded in order to reduce potential confounding. We used energetics data from these intervals to calculate the AUC and adjusted these for fasting levels. DIT, protein oxidation (OX_PRO_), carbohydrate oxidation (OX_CHO_), and fat oxidation (OX_FAT_) were calculated at each interval with the following equations:

(1) $$\mathrm{DIT}=\left(\frac{(\left(\mathrm{Postprandial}\;\mathrm{EE}\right)-(\mathrm{Resting}\;\mathrm{Metabolic}\;\mathrm{Rate}\times 217.5))}{\left(\mathrm{Resting}\;\mathrm{Metabolic}\;\mathrm{Rate}\;\times \;217.5\right)}\right)\;\times \;100$$

(2) $${\mathrm{OX}}_{\mathrm{PRO}}=\frac{\left(N_{2}\;\times \;6.26\right)}{0.996}$$

(3) $${\mathrm{OX}}_{\mathrm{CHO}}=\left(4.113{\;\times \;\mathrm{VCO}}_{2}\right)-\left(2.907{\;\times \mathrm{VO}}_{2}\right)-(3.75\;\times \;{\mathrm{OX}}_{\mathrm{PRO}})$$

(4) $${\mathrm{OX}}_{\mathrm{FAT}}=\left(1.689\;\times \;O_{2}\right)-\left(1.689\;\times \;{\mathrm{CO}}_{2}\right)\;-\;(0.324\;\times \;{\mathrm{OX}}_{\mathrm{PRO}})$$

The incremental area under the curve (iAUC) was calculated by the trapezoidal method. All statistical analyses were performed using JMP Pro 15.1.0 (SAS Institute, Cary, NC). For energetics responses (EE, CHO Ox, Fat Ox, and DIT) and group name (ghrelin, TAG, insulin, and glucose) iAUC’s, a linear mixed model was used assigning treatment (i.e., diet) as a fixed effect and participant as a repeated random effect across visits, with compound symmetry covariance structure; comparison among diets used the Tukey-Kramer multi-comparison adjustment. To evaluate the immediate temporal response (0–240 min) to dietary fat source effects, a two-way interaction mixed ANOVA with repeated measures was performed to evaluate diet-by-time effects. Diet and time, and their interaction, were fixed effects. Time was the residual random effect for participants within visits as the subject of the repeated measure. An unequal variance compound symmetry for time points within visits was used. Tukey’s multiple comparison adjustment was used for post hoc tests of specific interest to determine differences between dietary fat types. Statistical significance of mean differences was taken as *p* < 0.05. Data are reported as the raw means ± standard deviations (SD) of the data.

## 3. Results

### 3.1. Participant Characteristics

Participant characteristics at screening are provided in Table 2. Participants ranged in age from 18 to 42 years with an average BMI of 27.7 ± 3.8 kg/m^2^ and mean percent body fat of 33.5 ± 8.2%. There were no differences in the body mass of each individual measured at admission into the metabolic unit (85.0 ± 13.3 kg, mean ± SD).

### 3.2. Postprandial Energy Expenditure

**Figure 2:**
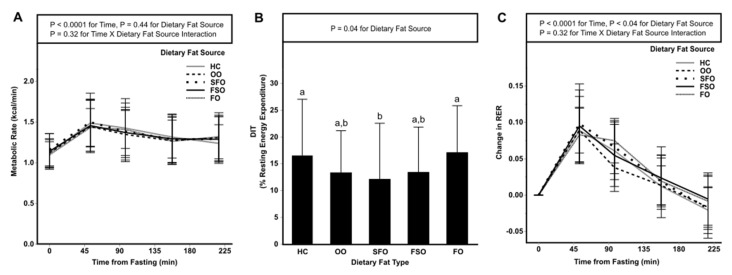
Metabolic rate (**A**), DIT (**B**), and RER (**C**) in an acute study of five dietary fat sources on satiety and energy expenditure in normal to obese participants. Values are the mean ± SD, *n* = 23 except HC, *n* = 20. Metabolic rate data (**A**) were compared using a linear mixed model was used with Tukey-Kramer multi-comparison adjustment. The DIT data (**B**) were compared using a linear mixed model was used with a Tukey-Kramer multi-comparison adjustment. The RER data (**C**) were compared using a two-way interaction mixed ANOVA with repeated measures was performed with a Tukey’s multiple comparison adjustment. Labeled means for each treatment (**B**) without a common letter differ, *p* < 0.05. DIT, diet-induced thermogenesis; FO, fish oil; FSO, flaxseed oil; HC, heavy cream; OO, olive oil; RER, respiratory exchange ratio; SFO, sunflower seed oil.

We compared DIT, RER, OX_CHO_, and OX_FAT_ between dietary fat sources during the postprandial period for 240 min in 15 min intervals between blood draws (Figure 2, Table 3). Changes in energy expenditure following the test meal for the individual time points are depicted in Figure 2A. A time effect (*p* < 0.0001) was observed, but there were no differences between dietary fat sources. DIT differed between fat sources (*p* = 0.04), with HC and FO having a higher mean DIT (approximately 5%) than SFO (Figure 2B). There was an effect of time (*p* < 0.01) on the postprandial change in RER and an effect of dietary fat source (*p* = 0.04) in which OO was different than SFO (*p* = 0.02) and FSO (*p* = 0.004). Interaction of time and the dietary fat source was not observed. Overall energy expenditure, OX_FAT,_ and OX_CHO_ over the postprandial period did not differ by dietary fat source (Table 3).

### 3.3. Subjective Measures of Satiety and Energy Intake of a Subsequent Meal

**Figure 3:**
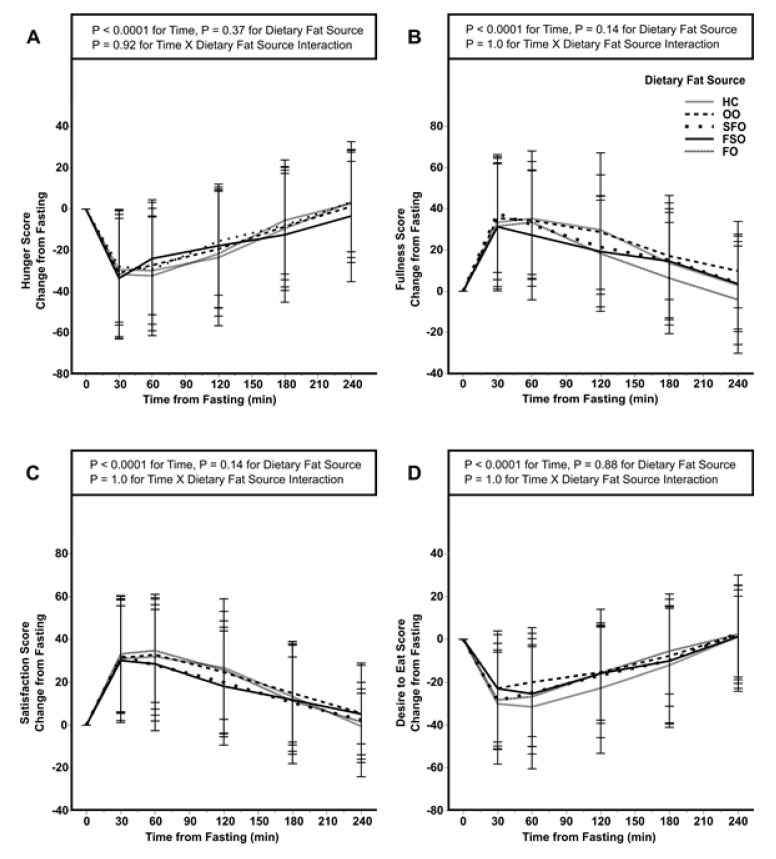
Hunger (**A**), fullness (**B**), satisfaction (**C**), and desire to eat (**D**) from an acute test of five dietary fat sources in normal to obese participants. Values are means ± SD; *n* = 23 except HC, *n* = 21. Data were compared using a two-way interaction mixed ANOVA with repeated measures with a Tukey’s multiple comparison adjustment. FO, fish oil; FSO, flaxseed oil; HC, heavy cream; OO, olive oil; SFO, sunflower seed oil.

We examined the influence of dietary fat sources upon the SMS endpoints of hunger, satisfaction, fullness, and desire to eat on a 100 mm visual analogue scale after each blood draw (Figure 3). As expected, the main effect of time was found following the meal for all SMS endpoints (*p* < 0.01); however, no dietary fat type-dependent differences or interactions were observed. There was no effect of fat type upon ESM (Supplementary Figure S1).

### 3.4. Postprandial Ghrelin, TAG, Insulin, and Glucose

**Figure 4:**
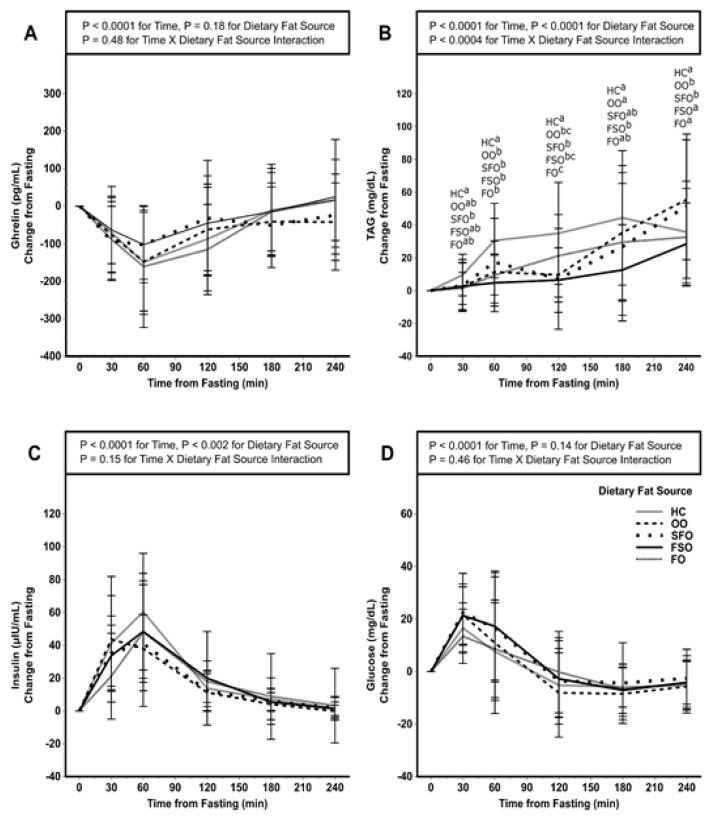
Ghrelin (**A**), TAG (**B**), insulin (**C**), and glucose (**D**) responses to an acute test of five dietary fat types on satiety and energy needs in normal to obese participants. Labeled means at each time point without a common letter differ, *p* < 0.05. A two-way interaction mixed ANOVA with repeated measures was performed with Tukey’s multiple comparison adjustment as a post hoc test. Values are means ± SD; *n* = 23 except for HC *n* = 21, insulin HC *n* = 19, and insulin FO *n* = 22. FO, fish oil; FSO, flaxseed oil; HC, heavy cream; OO, olive oil; SFO, sunflower seed oil.

Postprandial concentrations of ghrelin, TAG, insulin, and glucose relative to fasting levels are depicted in Figure 4 and Table 4. Postprandial ghrelin concentrations were affected by time (*p* < 0.0001) in which concentrations decreased after the consumption of each dietary fat until 60 min then rose until the 240 min time point (Figure 4A). However, there was no effect of dietary fat type nor was there an interaction between time and dietary fat type when comparing iAUC (Table 4). There was an effect of time on the postprandial change in TAG response (*p* < 0.0001), an effect of the dietary fat source (*p* < 0.0001), and a significant interaction of time and dietary fat source (*p* < 0.0004) (Figure 4B). Higher concentrations with HC compared to the other fat sources were observed at early time points; whereas, differences, notably lower TAG concentrations following the OO meal were evident starting at 180 min. The iAUC for the postprandial changes in TAG (Table 4) was greater (*p* < 0.01) for the HC meal compared to meals with FSO and FO.

Time affected postprandial insulin responses (*p* < 0.01), and there was an effect of dietary fat source (*p* = 0.002) resulting from a difference between the HC and FO responses over time (*p* = 0.02) (Figure 4C). Interaction of time and the dietary fat source was not observed. There were no differences in the iAUC for insulin between dietary fat sources (Table 4). There was a main effect of time (*p* < 0.0001) for glucose (Figure 4D). We observed no effect of dietary fat source on the change in glucose from fasting. The glucose iAUC was not affected by the dietary fat source for the 240 min postprandial period (Table 4).

## 4. Discussion

The objective of this work was to evaluate the roles of dietary fats consisting of fatty acids with differing chain lengths and degrees of saturation in modulating postprandial energy expenditure and satiety in a concurrent manner. Our data demonstrate a difference in DIT with SFO, containing the *n*-6 PUFA LA, having a lower DIT than HC and FO. No differences in satiety responses occurred between fat sources containing fatty acids encompassing a wide spectrum of saturation and chain lengths. The dietary fat source had little effect on acute postprandial responses for ghrelin, glucose, and insulin. HC intake resulted in a greater postprandial lipemic response than most of the other fat types in agreement with other studies [33,34,35], indicating that differential metabolism of the test fats did occur.

We evaluated the effects of dietary fat sources on energy expenditure and substrate utilization over a 240 min postprandial period and observed that SFO, as a source of LA, induced a lower amount of DIT than HC, the SFA source, and FO, a source of LCn-3. Our results contrast with the data reported by Clevenger and colleagues in which dietary fat sources with elevated contents of SFA, MUFA, or 18-carbon PUFA did not influence acute energetic responses in obese women [12], and are in contrast to studies demonstrating elevated DIT after MUFA and PUFA intake compared to SFA when women or men with a healthy body weight were studied [11,16]. We note that in our study, the *n*-3 and *n*-6 PUFA oils sources were evaluated separately, as FSO and SFO, respectively, whereas some studies used a mixture of *n*-3 and *n*-6 PUFA in oil form or provided *n*-3 PUFA in the form of nuts [11,12,16]. We observed no differences in energetic endpoints between the 18-carbon *n*-3 and *n*-6 PUFA oils sources, FSO and SFO, respectively. These data agree with those of Jones and colleagues comparing SFA and FSO but do differ in that an elevated DIT when using olive oil as a source of OA vs. ALA from flaxseed oil in healthy weight men [13].

Results from mechanistic studies vary with respect to the differential oxidation of dietary fatty acids. DeLany and colleagues, using ^13^C-labeled fatty acids, demonstrated that dietary ALA is oxidized at a higher rate than LA, the SFA 16:0 and 18:0, and the MUFA 18:1*n*-9 [18]. Other studies of fatty acid oxidation by McCloy and colleagues, using ^13^C-labeled fatty acids, demonstrated a similar degree of oxidation of dietary 18:1*n*-9 and ALA and a lesser degree of oxidation of LA due in part to a higher partitioning of LA into phospholipids and cholesteryl esters [36]. We are cognizant, however, that (1) our studies used commercially-available oil sources that, while high in a particular fatty acid type, still contained a variety of fatty acid types, and that (2) analysis of oxidation rates of labeled fatty acids is not directly comparable to the measurement of energy expenditure resulting from intake of dietary fats given the sensitivity of labeled isotope measurements.

An important factor in the role of dietary fat source on mediating long-term energy balance is the potential to affect postprandial satiety. However, we found no differences in SMS or ESM between dietary fat sources. Previously published literature examining the effects of fatty acids in meals and in intestinal infusion models have produced mixed results with SMS and ESM. Ileal infusions of a MUFA (18:1*n*-9) and of LA increase fullness and decrease hunger compared to 18:0, but another study of duodenal infusions did not show differences in SMS between fat types although an LA-rich infusion reduced subsequent food intake compared to a MUFA-rich infusion [19,20]. In acute feeding studies using similar energy content and fat loads to our experimental design, no differences among dietary fat sources have been observed in SMS or ESM [16,21,37]. However, investigations using high energy and fat content (691–1358 kcal and 55–70% fat) have shown varying effects of dietary fat sources on SMS [23,38,39]. Our meals contained from 456–517 kcal with 30 g of fat which may align more closely with normal intakes, but which may not be sufficient to determine differences in SMS. Taken together, these data suggest that detecting differences between dietary fat sources in their effects on SMS is influenced by both the amount of food relative to daily energy needs and to the percentage of energy from fat in a meal.

There are contrasting data regarding the effects of SFA upon satiety endpoints compared to MUFA and PUFA. The SFA source may contribute to this heterogeneity. Studies using 18:0 (stearic acid) as the primary SFA source demonstrate greater satiety responses with MUFA and PUFA vs. SFA [19,20,38]. However, with the exception of Kozimor and colleagues, most studies using dairy-derived fat (comprising a mixture of SFA types) or a mixture of dietary SFA sources with medium and long chain SFA show no differences in SMS like hunger and fullness [16,21,22,23,40]. Similarly, the predominant fatty acid in our HC intervention was 16:0 with smaller concentrations of 14:0 and 18:0. One study has reported no difference in satiety endpoints based on SFA chain length while another recent study indicates that differences in the satiety effects of medium chain SFA sources exist [41,42]. Differences in SFA-type responses may arise from the differential absorption of specific SFA (e.g., 12:0 vs. 16:0 vs. 18:0) or metabolism of the dietary TAG species [9,43,44].

Subjective satiety is in part mediated by peripheral hormonal control. Ghrelin is a 28-amino acid orexigenic peptide secreted primarily from the stomach and its secretion is blunted in response to food intake [45,46]. Fat absorption suppresses ghrelin concentrations, and dietary fat sources have been reported to influence the magnitude of ghrelin suppression [47,48,49]. While we observed no effect of dietary fat type on postprandial suppression of serum ghrelin, previous results in obese females show a greater suppression of postprandial ghrelin from PUFA- and MUFA-rich high-fat meals compared to SFA-rich meals [39]. These contrasting results may be explained by the inclusion of men in this study; however, acute intake of SFA is reported to have no effect on ghrelin in lean men [50].

Our data demonstrate that PUFA type may modify postprandial energy expenditure. It is well-known that *n*-3 and *n*-6 PUFA have divergent physiologic effects [51]. Most studies investigating the effects of dietary PUFA on satiety and energetic responses have used either LA-containing or a mixture of LA- and ALA-containing oils, but only a limited number of studies have compared LA to ALA [11,12,13,16,17]. A large number of studies report LCn-3-induced increases in fat oxidation in rodents and humans following long-term supplementation, and hence four hours may be insufficient time for LCn-3 to increase β-oxidation through hepatic gene expression as has been proposed (see reviews [52,53,54]). Our findings are important given that LCn-3 supplements are widely available. While our study used FO as a commonly used source of LCn-3, FO contains a variety of fatty acid types. Subsequent studies are needed with more highly refined forms of LCn-3.

This study has limitations. Body size and composition may affect postprandial SMS and ESM when using isocaloric meals for all body sizes. Drawing blood affects energy expenditure during and in the minutes following the blood draw. To reduce the impacts of blood sampling, we analyzed gas exchange between blood draws when there was no participant activity. While dietary lipid is not commonly consumed in a liquid meal format, we chose liquid meals to limit potential food matrix effects which could affect fat absorption, subsequent energy expenditure, and satiety. Although there may be confounds related to food matrix effects with the use of HC which contains milk fat globule membrane, HC complied with the liquid meal format versus using other, solid sources of SFA. We selected men and only women on hormonal contraceptives to reduce metabolic fluctuation during the menstrual cycle, but sex differences may still exist in satiety and energy metabolism [55]. We acknowledge that our acute study does not necessarily reflect on long-term satiety/weight management. Although the liquid meals were formulated to contain 30 g of fat, there were differences in the total energy content (6 kcal to 61 kcal) due to the matrices of the fat sources. Based upon the meta-regression analysis of Quatela and colleagues, these differences would impact DIT by no more than 1% [56]. While carryover is a potential concern, carryover effects were minimized by (1) provision of only one, single meal test, (2) provision of the lead-in diet three days prior to testing, (3) randomization of treatment, and (4) a minimum of 1-week washout. The differences in BMI may underlie some of the differences in results. The BMI of our participants ranged from normal weight to obese (19.9–34.0 kg/m^2^) but was mainly that of overweight to obese (27.7 ± 3.8 kg/m^2^).

Strengths of this study include the extensive fat source comparisons upon energetic and satiety measures. The use of a controlled, lead-in diet for three days with equal proportions of SFA, MUFA, and PUFA prior to the test day provided a control for the potential metabolic adaptations to fat intake that have been demonstrated in other studies [40,57]. The whole room calorimeter assesses gas exchange continuously enabled the analysis of energy expenditure and substrate oxidation without the potential confound of a face mask or hood used with metabolic carts.

## 5. Conclusions

Our data demonstrate some differences in DIT, but not substrate oxidation and satiety, following acute meal challenges with dietary fat sources comprising a broad spectrum of chain length and degree of saturation. Differences in postprandial TAG in response to dietary fat type warrant further investigation with other sources of SFA. Further studies are needed to identify the relationship between dietary fat sources and energy expenditure following the intake of subsequent meals and to determine the influences of different food matrices.

## Figures and Tables

**Table 1:** Macronutrient content of the liquid test meals consumed by participants in an acute study of fat source on energy expenditure and satiety ^1^.

Test Meal	Amount (g)	Energy (kcal)	Protein (g)	Carbohydrate (g)	Fat (g)
**Heavy Cream**					
Frozen Strawberries	100	35	0.4	9.1	0.1
Orange Sherbet	100	128	1.1	31.4	0.0
Skim Milk	100	34	3.4	5.0	0.1
Heavy Cream	86	287	1.8	2.4	30.0
Total	386	484	6.7	47.9	30.2
**Olive Oil**					
Strawberries	100	35	0.4	9.1	0.1
Orange Sherbet	100	128	1.1	31.4	0.0
Skim Milk	100	34	3.4	5.0	0.1
Olive Oil	30	265	0.0	0.0	30.0
Total	330	462	4.9	45.5	30.2
**Sunflower Oil**					
Strawberries	100	35	0.4	9.1	0.1
Orange Sherbet	100	128	1.1	31.4	0.0
Skim Milk	100	34	3.4	5.0	0.1
Sunflower Oil	30	259	0.0	0.0	29.4
Total	330	456	4.9	45.5	29.6
**Flaxseed Oil**					
Strawberries	100	35	0.4	9.1	0.1
Orange Sherbet	100	128	1.1	31.4	0.0
Skim Milk	100	34	3.4	5.0	0.1
Flaxseed Oil	30	270	0.0	0.0	30.0
Total	330	467	4.9	45.5	30.2
**Fish Oil**					
Strawberries	100	35	0.4	9.1	0.1
Orange Sherbet	100	128	1.1	31.4	0.0
Skim Milk	100	34	3.4	5.0	0.1
CorOmega Omega3 Squeeze	40	320	0.0	0.0	29.6
Total	340	517	4.9	45.5	29.8

^1^ The nutrient content for this table was calculated using the in-house nutrient database (GRAND). GRAND Database nutrient values for this report were from Release 27 of the USDA Nutrient Database for Standard Reference [29]. For CorOmega squeeze, nutrient contents were taken from the nutrient facts panel of the product.

**Table 2:** Baseline characteristics of study participants consuming five test fats in an acute study of fat source on energy expenditure and satiety ^1^.

Age (years)	25.7 ± 6.6
Body fat (%)	33.5 ± 8.2
Body mass index (kg/m^2^)	27.7 ± 3.8
Fat free mass (kg)	52.2 ± 10.1

^1^ Values are means ± SD, *n* = 23.

**Table 3:** Energy expenditure, fat oxidation, and carbohydrate oxidation for the 240 min (AUC) postprandial period of participants consuming five test meals containing one of five fats in an acute study of fat source on energy expenditure and satiety ^1^.

Endpoint	HC ^2^	OO	SFO	FSO	FO	*p* ^3^
EE (kcal)	293 ± 55	286 ± 60	292 ± 64	289 ± 58	288 ± 49	0.27
OX_FAT_ (g)	10.3 ± 12.2	7.9 ± 10	5.6 ± 14.2	7.3 ± 10.7	8.7 ± 12.1	0.31
OX_CHO_ (g)	9.5 ± 6.5	6.5 ± 6.1	8.8 ± 8.5	9.4 ± 6.6	8.2 ± 6.8	0.31

^1^ Values are means ± SDs. *n* = 23 except for HC, *n* = 20; ^2^ EE, energy expenditure; FO, fish oil; FSO, Flaxseed oil; HC, heavy cream; OXCHO, carbohydrate oxidation; OXFAT, fat oxidation; OO, olive oil; SFO, sunflower seed oil; ^3^ A linear mixed model was used with Tukey-Kramer multi-comparison adjustment.

**Table 4:** Incremental area under the curve for serum endpoints of participants consuming five test meals containing one of five fats in an acute study of fat source on energy expenditure and satiety.

Endpoint	HC	OO	SFO	FSO	FO	*p* ^3^
Ghrelin (pg/mL × h)	−249 ± 428 ^1^	−242 ± 344	−201 ± 338	−189 ± 389	−290 ± 379	0.86
TAG (mg/dL × h)	126 ± 88 ^a,2^	81 ± 62 ^ab^	75 ± 101 ^ab^	49 ± 69 ^b^	62 ± 77 ^b^	<0.01
Insulin (μIU/mL × h)	78 ± 31	67 ± 31	70 ± 48	79 ± 51	64 ± 28	0.11
Glucose (mg/dL × h)	−3 ± 26	1 ± 36	16 ± 33	14 ± 25	1 ± 40	0.13

^1^ Values are means ± SDs. *n* = 23 except for HC, *n* = 21, HC for insulin *n* = 19, FO for insulin *n* = 22. Values are baseline subtracted; ^2^ Values with different superscript letters are significantly different. FO, fish oil; FSO, flaxseed oil; HC, heavy cream; OO, olive oil; SFO, sunflower seed oil; TAG, triacylglycerol; ^3^ A two-way interaction mixed ANOVA with repeated measures was performed. Tukey’s multiple comparison adjustment was used for the post hoc test.

## Data Availability

Data will be curated at https://data.nal.usda.gov/.

## Supplementary Materials

The following are available online at https://www.mdpi.com/article/10.3390/nu13082615/s1, Figure S1: Energy of a subsequent meal from participants (*n* = 23) 5 h after consuming one of 5 liquid test meals in an acute study of fat source on energy expenditure and satiety; Table S1: Treatment order and the number of subjects completing each treatment order in a study of the acute effects of 5 dietary fat sources on satiety and energy expenditure; Table S2: Nutrient composition of the 3-day lead-in diet based on 2000 kcal; Table S3: Fatty acid content of the test meals consumed by participants consuming 5 test meals containing one of 5 fats in an acute study of fat source on energy expenditure and satiety; Table S4: Nutrient content of the post intervention meal consumed ad libitum used to determine the energy intake of a subsequent meal.

## Abbreviations

ALA: alpha-linolenic acid; ANOVA, analysis of variance; AUC, area under the curve; CHO, carbohydrate; DIT; diet-induced thermogenesis, en, energy; ESM, energy intake of a subsequent meal; FO, fish oil, FSO, flaxseed oil; GFHNRC, Grand Forks Human Nutrition Research Center; HC, heavy cream; iAUC, incremental area under the curve; LA, linoleic acid; LCn-3, long-chain *n*-3 polyunsaturated fatty acids; MUFA, monounsaturated fatty acids; OA, oleic acid; OO, olive oil; OX_CHO_, carbohydrate oxidation rate; OX_FAT_, fat oxidation rate; OX_PRO,_ protein oxidation rate; PUFA, polyunsaturated fatty acids; REE, resting energy expenditure; RMR, resting metabolic rate; SFA, saturated fatty acids; SFO, sunflower oil; SMS, subjective measures of satiety; TAG, triacylglycerol; VLDL, very low density lipoprotein.
