# Supplementary material for: Dietary Fat Chain Length, Saturation, and PUFA Source Acutely Affect Diet-Induced Thermogenesis but Not Satiety in Adults in a Randomized, Crossover Trial
Source: Nutrients. 2021 Jul 29;13(8):2615. doi: 10.3390/nu13082615 (PMC8402189; doi:10.3390/nu13082615)
Supplement: Supplementary file 1 [file nutrients-13-02615-s001.zip › nutrients-1230498-supplementary.pdf]

Supplementary Table S1. Treatment order and the number of subjects completing each treatment order in a study of the acute effects of 5 dietary fat sources on satiety and energy expenditure.

Order[1]	Number of completers
ABECD	3
AEBDC	1
BACED	2
BCADE	2
BECAD	1
CBDAE	2
CDBEA	3
DCEBA	3
DECAB	2
EADBC	2
EDACB	2
	23

[1]A, Olive oil; B, flaxseed oil; C, sunflower seed oil; D, heavy cream; E, fish oil

Supplementary Table S2 Nutrient composition of the 3-day lead-in diet based on 2000 kcal.

Nutrient content	3-day average		
Energy (kcal)	2003	±	5
Protein (g)	77	±	0
Carbohydrate (g)	256	±	1
Fat (g)	80	±	0
Protein% en	15	±	0
Carbohydrate % en	50	±	0
Fat % en	35	±	0
SFA (g)	24	±	0
MUFA (g)	24	±	0
PUFA (g)	24	±	0
SFA% en	11	±	0
MUFA% en	11	±	0
PUFA% en	11	±	0
Dietary Fiber (g)	26	±	6
**Minerals**			
Calcium (mg)	780	±	242
Copper (mg)	1.2	±	0.2
Iron (mg)	21	±	7
Magnesium (mg)	317	±	60
Manganese (mg)	4.1	±	1.6
Phosphorus (mg)	1183	±	160
Zinc (mg)	11	±	2
Potassium (mg)	3014	±	445
Sodium (mg)	2575	±	711
**Water-soluble vitamins**			
Vitamin C (mg)	175	±	79
Thiamin (mg)	1.8	±	0.5
Riboflavin (mg)	1.9	±	0.4
Niacin (mg)	29	±	6
Vitamin B6 (mg)	2.2	±	1.0
Folate (μg)	737	±	287
Vitamin B12 (μg)	4.0	±	1.9
**Fat-soluble vitamins**			
Vitamin A (μg)	1100	±	405
Vitamin D (μg)	3.0	±	1.5
Vitamin E (mg)	13	±	2

The nutrient content for this table was calculated using the in-house nutrient database (GRAND). GRAND Database nutrient values for this report were from Release 27 of the USDA Nutrient Database for Standard Reference [1].

Supplementary Table S3 Fatty acid content of the test meals consumed by participants consuming 5 test meals containing one of 5 fats in an acute study of fat source on energy expenditure and satiety.[1]

Fatty Acid	HC[2] (g)	OO (g)	SFO (g)	FSO (g)	FO (g)
6:0[3]	0.32 ± 0.01	ND	ND	ND	ND
8:0	0.33 ± 0.00	ND	ND	ND	ND
10:0	0.83 ± 0.02	ND	ND	ND	ND
11:0	0.09 ± 0.00	ND	ND	ND	ND
12:0	1.00 ± 0.01	ND	ND	ND	0.06 ± 0.00
14:0	3.75 ± 0.14	ND	ND	ND	<LOQ
15:0	0.38 ± 0.00	ND	ND	ND	<LOQ
16:0	10.72 ± 0.08	3.07 ± 0.06	1.99 ± 0.07	1.81 ± 0.05	5.51 ± 0.38
17:0	0.25 ± 0.00	0.06 ± 0.00	<LOQ	0.04 ± 0.00	0.16 ± 0.01
18:0	3.35 ± 0.05	1.20 ± 0.03	1.10 ± 0.06	1.63 ± 0.02	1.20 ± 0.08
20:0	0.06 ± 0.00	0.14 ± 0.01	0.08 ± 0.01	0.08 ± 0.01	0.16 ± 0.01
22:0	ND	0.08 ± 0.01	0.21 ± 0.01	0.07 ± 0.00	<LOQ
Total SFA	21.09	4.55	3.38	3.64	7.10
12:1	0.07 ± 0.00	ND	ND	ND	ND
16:1 (n-7) E	ND	ND	ND	ND	0.21 ± 0.01
16:1 (n-7) Z	0.49 ± 0.01	0.15 ± 0.01	<LOQ	<LOQ	2.64 ± 0.16
18:1 (n-7) Z	0.19 ± 0.03	0.52 ± 0.04	<LOQ	0.18 ± 0.03	0.98 ± 0.19
18:1 (n-9) E	0.63 ± 0.03	ND	ND	ND	<LOQ
18:1 (n-9) Z	6.33 ± 0.10	12.9 ± 0.09	7.90 ± 0.04	5.18 ± 0.01	3.35 ± 0.17
20:1 (n-9) Z	<LOQ	0.13 ± 0.01	0.05 ± 0.00	<LOQ	0.59 ± 0.03
24:1 (n-9) Z	ND	ND	ND	ND	0.15 ± 0.01
Total MUFA	7.71	13.70	7.95	5.36	7.92
18:2 (n-6)	0.83 ± 0.05	10.40 ± 0.09	18.43 ± 0.11	4.82 ± 0.05	0.65 ± 0.04
18:3 (n-6)	<LOQ	0.06 ± 0.00	ND	<LOQ	<LOQ
20:2 (n-6)	ND	ND	ND	ND	0.97 ± 0.07

20:3 (n-6)	ND	ND	ND	ND	0.04 ± 0.00
20:4 (n-6)	ND	ND	ND	ND	0.34 ± 0.02
18:3 (n-3)	<LOQ	1.34 ± 0.04	<LOQ	15.58 ± 0.08	<LOQ
20:5 (n-3)	ND	ND	ND	ND	5.77 ± 0.25
22:5 (n-3)	ND	ND	ND	ND	0.61 ± 0.05
22:6 (n-3)	ND	ND	ND	ND	3.84 ± 0.19
Total PUFA	0.83	11.80	18.43	20.40	12.22
Total	29.63	30.05	29.76	29.40	27.24

[1]Values are means ± SD; n = 3 separate meal preparations.

[2]; FO, fish oil; FSO, flaxseed oil; HC, heavy cream; LOQ, limit of quantitation; MUFA, monounsaturated fats; ND, below the limits of detection; OO, olive oil; PUFA, polyunsaturated fats; SFA, saturated fatty acids. SFO, sunflower seed oil.

[3]Quantified by gas chromatography with flame ionization detection.

Supplementary Table S4. Nutrient content of the post intervention meal consumed *ad libitum* used to determine the energy intake of a subsequent meal

Macronutrients	Post intervention meal
Energy (kcal)	1785
Protein (g)	108
Carbohydrate (g)	166
Fat (g)	75
Protein (% en)	24.5
Carbohydrate (% en)	37.5
Fat (% en)	38.0
SFA (g)	27
MUFA (g)	21
PUFA (g)	16
SFA (% en)	14
MUFA (% en)	11
PUFA (% en)	8
Dietary Fiber (g)	8
**Minerals**	
Calcium (mg)	700
Copper (mg)	1.0
Iron (mg)	13
Magnesium (mg)	198
Manganese (mg)	1.9
Phosphorus (mg)	1323

Zinc (mg)	9
Potassium (mg)	1028
Sodium (mg)	4574
**Water-soluble vitamins**	
Vitamin C (mg)	0.3
Thiamin (mg)	1.7
Riboflavin (mg)	1.4
Niacin (mg)	37
Vitamin B_6 (mg)	1.4
Folate, (μg)	729
Vitamin B_{12} (μg)	1.8
**Fat-soluble vitamins**	
Vitamin A (μg)	377
Vitamin D (μg)	1.7
Vitamin E (mg)	4.9

The nutrient content for this table was calculated using the in-house nutrient database (GRAND). GRAND Database nutrient values for this report were from Release 27 of the USDA Nutrient Database for Standard Reference [1].

Supplementary Figure S1. Energy intake of a subsequent meal from participants (n = 23) 5 hours after consuming one of 5 liquid test meals in an acute study of fat source on energy expenditure and satiety.

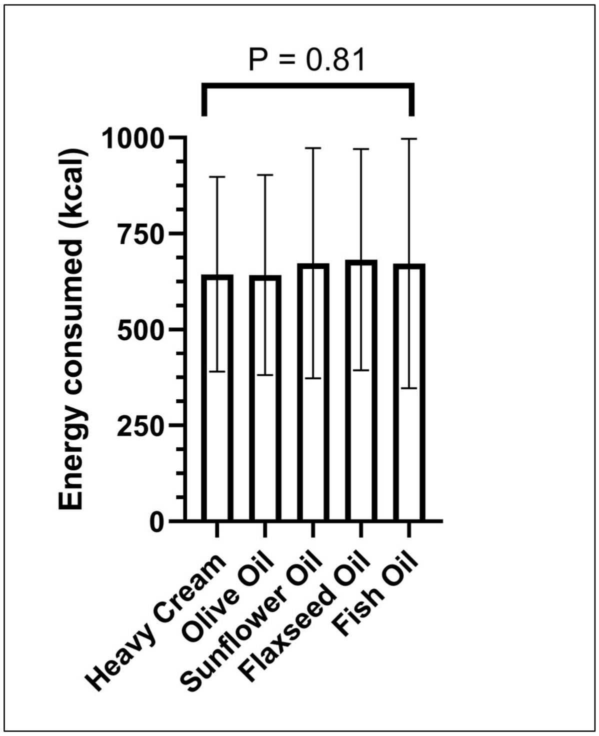

References

1. USDA. USDA National Nutrient Database for Standard Reference, Release 27. U.S. Department of Agriculture, Agricultural Research Service http://www.ars.usda.gov/ba/bhnrc/ndl: 2014.
